# A Congeneric Comparison Shows That Experimental Warming Enhances the Growth of Invasive *Eupatorium adenophorum*

**DOI:** 10.1371/journal.pone.0035681

**Published:** 2012-04-20

**Authors:** Wei-Ming He, Jing-Ji Li, Pei-Hao Peng

**Affiliations:** 1 State Key Laboratory of Vegetation and Environmental Change, Institute of Botany, the Chinese Academy of Sciences, Beijing, China; 2 Department of Landscape Architecture, Chengdu University of Technology, Chengdu, China; DOE Pacific Northwest National Laboratory, United States of America

## Abstract

Rising air temperatures may change the risks of invasive plants; however, little is known about how different warming timings affect the growth and stress-tolerance of invasive plants. We conducted an experiment with an invasive plant *Eupatorium adenophorum* and a native congener *Eupatorium chinense*, and contrasted their mortality, plant height, total biomass, and biomass allocation in ambient, day-, night-, and daily-warming treatments. The mortality of plants was significantly higher in *E. chinense* than *E. adenophorum* in four temperature regimes. *Eupatorium adenophorum* grew larger than *E. chinense* in the ambient climate, and this difference was amplified with warming. On the basis of the net effects of warming, daily-warming exhibited the strongest influence on *E. adenophorum*, followed by day-warming and night-warming. There was a positive correlation between total biomass and root weight ratio in *E. adenophorum*, but not in *E. chinense*. These findings suggest that climate warming may enhance *E. adenophorum* invasions through increasing its growth and stress-tolerance, and that day-, night- and daily-warming may play different roles in this facilitation.

## Introduction

Air temperature is a fundamental condition limiting communities, and changes in temperatures may influence the performance of individual species [1]–[3]. Global surface temperatures are projected to increase by 1.8–4.0°C by the end of this century and climate warming exhibits uncertainty [4]. Recent studies suggest that warming timing plays important roles in plant ecology. For example, day-, night- and daily-warming can differentially shift the benefits of clonal integration [5], and affect the carbon budgets of temperate steppe ecosystems [6]. However, little is known about the effects of different warming timings on invasive plants.

In general climate warming exhibits direct and indirect effects on plants. Warming affects plants' physiological processes such as photosynthesis and respiration directly, altering source-sink relations of photosynthesis [7]–[9]. On the other hand, warming can change microclimates and soil water regimes, resulting in multiple stresses [1], [2]. It is likely that different climate warming timings pose differential effects on source-sink relationships and multiple stresses, subsequently influencing the growth and stress-tolerance of plants. It is still poorly known, to our knowledge, that how day-, night- and daily-warming influence these two aspects.

Invasive plants are currently expanding regionally and globally [10]. This raises concerns over how climate warming influences the risks of invasive plants [11], [12]. Although evidence from models suggests that climate warming tends to facilitate the invasion of exotic plants [13], [14], experimental evidence is still limited [15]. In contrast, there is also evidence that warming may cause declines of populations of invasive plants [16]. Successful invasive plants are usually characterized by faster growth or higher tolerance [17], [18]. If different warming timings can pose contrasting consequences for source-sink relationships and multiple stresses, these subsequent changes may modulate the invasion of exotic plants through changing their growth or stress-tolerance.

Comparisons of native versus invasive congeners are highly valuable to predict the risks of plant invaders in new ranges, particularly when coupled with a variety of experimental manipulations [19]. *Eupatorium adenophorum*, native to Central America, is a noxious invasive plant worldwide [20]. This species invaded southwest China in the 1940s from Burma and Vietanm and is expanding rapidly due to low herbivore loads [21], [22]. *Eupatorium adenophorum* usually invades roadside, abandoned fields, agricultural fields, pastures, disturbed forests and limestone shrubs, and replaces local dominant native plants or even forms almost monocultures in some habitats [21]. *Eupatorium chinense* is among the local dominant plants and often occurs in the understory and edge of forests, shrubs and grasslands. This species' distribution is currently declining rapidly due to the invasion of *E. adenophorum* [21]. Both species are 1–2 m tall perennial forbs. Air temperatures are predicted to increase by 1.2–3.3°C by the end of this century in Chinese subtropical regions [23]. These situations set up a unique stage for understanding the growth of *E. adenophorum* under climate warming by comparing its performance with *E. chinense*.

The central goal of this study was to explore how different warming timings affect the growth and stress-tolerance of *E. adenophorum* and *E. chinense* in subtropical regions, where rainfall is plentiful and soils are fertile. We examined whether day-, night-, and daily-warming favor the growth of *E. adenophorum* over *E. chinense* and how these warming timings alter the tolerance of plants to cope with multiple stresses resulting from warming.

## Materials and Methods

We conducted an experiment at our field station in Chengdu (30.67°N, 104.06°E) using plants of *E. adenophorum* and *E. chinense* grown from seeds, which were collected from the *E. adenophorum*-invaded communities with *E. chinense*. Because this area has long been invaded by *E. adenophorum*, no specific permits were required for this study. All plants were grown alone in 3 L pots (i.e. upper diameter: 20 cm; lower diameter: 10 cm; height: 17 cm) filled with the local soils from the same community as seeds. On April 15, 2010, 10 similar plants per species were selected to be subjected to each of the four warming treatments: control (ambient), day-warming (7:00–19:00), night-warming (19:00–7:00), and daily-warming (24 h). Each warming treatment was heated during the experiment with a HS-2408 infrared radiator (Kalglo Electronics, Bethlehem, PA, USA) that was suspended 1.5 m above the soil surface. One ‘dummy’ heater with the same shape and size as the infrared radiator was used to simulate the shading effect of the infrared radiator in the unwarmed control. This heating approach increased the air temperatures surrounding the target plants by about 2°C (ranging between 1.5–2.5°C), which is in the range of air temperatures projected by previous studies [4], [20]. The other conditions, including irradiance, rainfall and soil, were similar to those in *E. adenophorum*-invaded communities, allowing us to mimic the field situations and to test the effects of climate warming on the performance of two congeners.

For each species we initially planted 40 individuals from seeds. After losses from mortality, replication varied from 5 to 10 individuals for the day- and night-warming treatments; in the daily-warming treatment, the plants of *E. chinense* gradually died and were gone before July 2010, and eight individuals of *E. adenophorum* survived. This experiment ran from April 15, 2010 to September 25, 2010, which roughly corresponds to the growing seasons in southwestern China. During the course of the experiment, the total rainfall was about 600 mm, and no additional water and nutrients were supplied. To minimize the effect of herbivores, we sprayed insecticides if necessary. At the end of the experiment, the height of each plant in a pot was determined with a ruler, and then all plants were harvested, washed, and separated into shoot and roots. These materials were oven-dried for 48 h at 75°C and weighed. To quantify the effects of experimental warming on plant height and biomass production, we calculated the relative change in plant height and total biomass as: (*V_w_*−*V_a_*)/*V_a_*×100%, where *V_w_* is the plant height or total biomass of a plant grown in a given warming treatment and *V_a_* is the mean plant height or mean biomass of plants grown in the control treatment. Root weight ratio (RWR) was calculated as the ratio of root biomass to the whole-plant biomass.

For the plants of *E. adenophorum* and *E. chinense* grown in the control temperature, we used the General Linear Model, where species identity was treated as a fixed factor, to test whether there were differences in plant height, total biomass, and RWR between invasive and native congeneric species. For a given species, there were four different air temperatures (i.e. ambient, day-warming, night-warming, and daily-warming) so that we used the General Linear Model, where temperature regime was treated as a fixed factor, to test the effects of different warming treatments on plant height, total biomass, and RWR. We compared mean responses to warming (i.e. changes in both plant height and total biomass with warming) between *E. adenophorum* and *E. chinense* using a one-tailed *t*-test. Pearson correlation coefficients were calculated to test the correlations between total biomass and RWR. All statistical analyses were carried out using SPSS 15.0 (SPSS Inc., Chicago).

## Results

**Figure 1:**
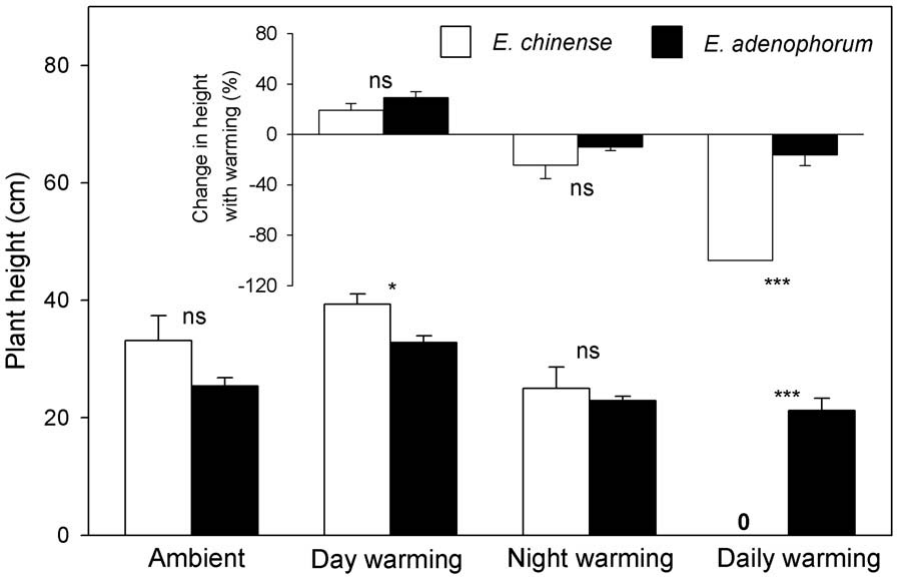
Plant height of the invasive plant *Eupatorium adenophorum* and the native plant *Eupatorium chinense* grown in four different air temperatures, and changes in plant height with warming for both species (embedded smaller panel inside). Data are means+1 SE. ns = not significant; * *P*<0.05; *** *P*<0.001.

**Figure 2:**
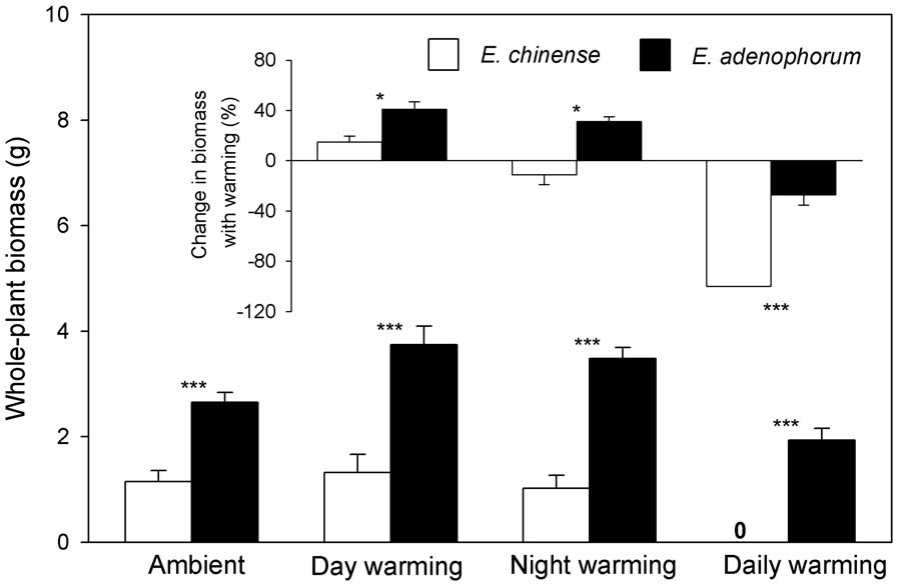
Whole-plant biomass of the invasive plant *Eupatorium adenophorum* and the native plant *Eupatorium chinense* grown in four different air temperatures, and changes in whole-plant biomass with warming for both species (embedded smaller panel inside). Data are means+1 SE. * *P*<0.05; *** *P*<0.001.

**Figure 3:**
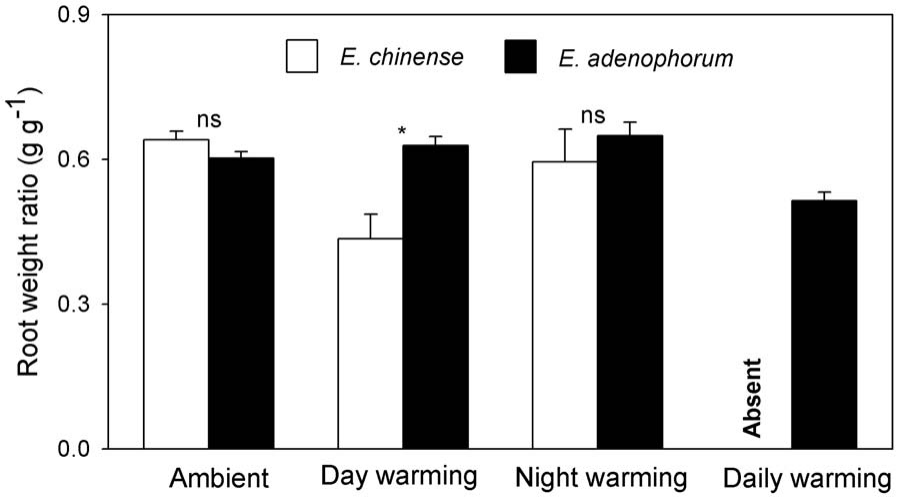
Root weight ratio of the invasive plant *Eupatorium adenophorum* and the native plant *Eupatorium chinense* grown in four different air temperatures. Data are means+1 SE. ns = not significant; * *P*<0.05.

In the control treatment, no plants died for *E. chinense* and *E. adenophorum*, both species shared similar plant height (Fig. 1; 33.1±4.2 (1 SE) cm vs 25.4±1.4 cm; *F*_1,18_ = 3.155, *P* = 0.078), *E. chinense* had much smaller biomass than *E. adenophorum* (Fig. 2; 1.15±0.21 g vs 2.65±0.19 g; *F*_1,18_ = 28.879, *P*<0.001), both species had similar root weight ratio (RWR) (Fig. 3; 0.64±0.02 vs 0.60±0.01; *F*_1,18_ = 2.487, *P* = 0.104). Thus, *E. adenophorum* possessed a greater canopy than *E. chinense*.

In the day-warming treatment, five out of 10 plants of *E. chinense* survived while all plants of *E. adenophorum* survived. Day-warming had no effects on plant height and total biomass of *E. chinense* (Figs. 1 & 2; all *P*>0.05), but allowed *E. adenophorum* plants to grow higher (Fig. 1; *F*_1,18_ = 15.596, *P* = 0.003) and to yield greater biomass (Fig. 2; *F*_1,18_ = 7.468, *P* = 0.019). In the day-warming treatment, *E. chinense* plants allocated less biomass to their roots (Fig. 3; *F*_1,13_ = 9.685, *P* = 0.014), but *E. adenophorum* plants did not have this response (Fig. 3; *F*_1,18_ = 1.127, *P* = 0.89).

In the night-warming treatment, seven out of 10 plants of *E. chinense* survived while all plants of *E. adenophorum* survived. Night-warming did not affect plant height of *E. chinense* and *E. adenophorum* (Fig. 1; all *P*>0.05). Night-warming had no effects on the biomass of *E. chinense* (Fig. 2; *F*_1,15_ = 2.827, *P* = 0.135), but allowed *E. adenophorum* to produce more biomass (Fig. 2; *F*_1,18_ = 4.871, *P* = 0.035). Night-warming did not affect biomass allocation in *E. adenophorum* and *E. chinense* (Fig. 3, all *P*>0.05).

In the daily-warming treatment, all plants of *E. chinense* died while only two out of 10 plants of *E. adenophorum* died. For *E. adenophorum*, daily-warming had no effects on its plant height and total biomass (Figs. 1 & 2; all *P*>0.05), but significantly decreased its RWR from 0.609±0.018 to 0.523±0.021 (Fig. 3; *F*_1,16_ = 4.937, *P* = 0.031).

The responses of plants to warming were species specific and heavily depended on warming treatments. Day-warming allowed strong growth of *E. chinense* and *E. adenophorum*, while night- and daily-warming had the opposite effect (the embedded smaller panel in Fig. 1). This height response to warming between *E. chinense* and *E. adenophorum* was significant only in the daily-warming treatment (*F*_1,16_ = 38.498, *P*<0.001), but not in the day- and night-warming treatments (all *P*>0.05) (the embedded smaller panel in Fig. 1). Day-warming facilitated two species to yield more biomass while daily-warming followed the opposite direction, and night-warming suppressed the growth of *E. chinense* but enhanced that of *E. adenophorum* (the embedded smaller panel in Fig. 1). This biomass response to warming between *E. chinense* and *E. adenophorum* was significant in the day-warming (*F*_1,13_ = 3.812, *P* = 0.045), night-warming (*F*_1,15_ = 9.238, *P* = 0.013), and diurnal-warming (*F*_1,16_ = 49.827, *P*<0.001) (the embedded smaller panel in Fig. 2).

Across three warming treatments, *E. adenophorum* had lower mortality than *E. chinense* (7±6% vs 60±21%; *F*_1,4_ = 5.953, *P* = 0.036), the biomass of *E. adenophorum* was greater than that of *E. chinense* (3.05±0.56 g vs 1.17±0.15 g; *F*_1,38_ = 2.972, *P* = 0.042), and both species shared similar RWR (0.597±0.042 vs 0.515±0.079; *F*_1,38_ = 0.953, *P* = 0.382). There was a significant correlation between biomass and RWR in *E. adenophorum* (*r* = 0.921, *P* = 0.040); in contrast, this correlation was not detected in *E. chinense* (*r* = −0.792, *P* = 0.209).

## Discussion

In this study we set up three different warming timings due to the uncertainty of climate warming. Our findings provide evidence that experimental warming allows the invasive plant *E. adenophorum* to outperform its congeneric native plant *E. chinense*, and that different warming timings exhibit contrasting effects on the growth and stress-tolerance of these two species. These findings also add to an understanding of the potential risks of plant invaders in the context of climate warming, particularly in those regions where rainfall and soil nutrients are plentiful and human disturbance is common.

Faster growth may be a general inherent trait of good invaders [15]. In our experiment, *E. adenophorum* plants grew larger than *E. chinense* plants in the control climate, indicating that the former has a fast-growing attribute. This phenomenon can be ascribed to higher fractions of leaf N to carboxylation and relatively high carbon gain per unit of N [21], [24]. Additionally, some invasive plants have access to more water than their congeneric natives, allowing them to achieve faster growth [25]. In contrast, it is likely that invasive and native congeners share similar resource use efficiency [26]. There were no differences in root weight ratio (RWR) between *E. adenophorum* and *E. chinense*, which is consistent with a previous study [27]. These findings also suggest that *E. adenophorum* plants have a larger canopy than *E. chinense* plants, allowing them to have a greater capacity to absorb light and shade other plant species.

Across three warming timings, *E. adenophorum* had a greater production potential and canopy and lower mortality than *E. chinense*, suggesting climate warming may be beneficial for *E. adenophorum* through favoring its growth, stress-tolerance and shading capacity over *E. chinense*. This superior performance of *E. adenophorum* in warming climates may be linked to its biogeographic niche. Specifically, *E. adenophorum* is native to warmer Central America and it thus may have been acclimated to warmer climate and exhibit higher temperature tolerance. This similar phenomenon has been found in recent studies [15], [28]. Because there are correlations between growth and competitive effects of invaders, their growth can predict their competitive effects [29]. Thus we propose a hypothesis that the growth advantage of *E. adenophorum* due to climate warming may allow it to become a good competitor.

Three warming timings had differential effects on the growth of *E. adenophorum* and *E chinense*, which can be attributed to changing source-sink relationships of photosynthesis. If greater carbohydrate consumption by plant respiration during the previous night can stimulate photosynthesis in the following day, then plant growth is enhanced, and vice versa [7]–[9]. Day-, night-, and daily-warming climates have different effects on leaf temperatures, subsequently influencing the source-sink relationships of photosynthesis [6], [8]. In this experiment, the day- and night-warming might induce the occurrence of photosynthetic overcompensation in *E. adenophorum*, while the daily-warming did not yield such an effect. Verlinden & Nijs (2010) found that invasive plants showed no response to daily-warming [15]. This is consistent with our findings.

Across all warming treatments, the survival rates of plants were greater in *E. adenophorum* than *E. chinens*. This survival also varied with warming timings. Carlos Cervera & Parra-Tabla (2009) found that invasive *R. nudiflora* had higher survival rates and extreme temperature tolerance than native *R. pereducta* [28], which is consistent with our findings. The daily temperatures were higher in the daily-warming than in the day- and night-warming, leading to heat shock. Thus this heat-shock tolerance may be lower in *E. chinense* than *E. adenophorum*, particularly in the daily-warming climate. *Eupatorium adenophorum* commonly invades open disturbed habitats [21], and its dense canopy can enable it to achieve higher survival and temperature tolerance by shedding its leaves. There is evidence that rising temperature of 2°C yields antagonistic effects on populations of two invasive plant species [16].

*Eupatorium adenophorum* is characterized by faster growth and greater canopy, which allows it to exhibit a higher potential for resource use and competition [17]. Future climate warming may enhance the invasion of *E. adenophorum* through increasing its growth and tolerance advantages, particularly in those habitats where the native plant *E. chinense* was a dominant species. Meanwhile, this facilitation strongly depends on warming timings. The success of invasions is closely linked to the competitive outcomes between invasive plants and native plants [30]. More studies are required to ascertain whether different climate warming timings can effectively tip the balance between *E. adenophorum* and its native plants in the field.
